# 2-year results of an RCT of 2 uncemented isoelastic monoblock acetabular components: lower wear rate with vitamin E blended highly cross-linked polyethylene compared to ultra-high molecular weight polyethylene

**DOI:** 10.1080/17453674.2020.1730073

**Published:** 2020-02-26

**Authors:** Joost H J van Erp, Julie R A Massier, Jelle J Halma, Thom E Snijders, Arthur de Gast

**Affiliations:** a Clinical Orthopedic Research Center—mN, Zeist, the Netherlands;; b Department of Orthopedic Surgery, Diakonessenhuis, Utrecht, the Netherlands

## Abstract

**Background and purpose** — The long-term survival of arthroplasty components may be limited by polyethylene wear-related problems such as periprosthetic osteolysis and aseptic loosening. Highly cross-linked polyethylene (HXLPE) blended with vitamin E was introduced to improve oxidative stability and to avoid long-term embrittlement. This study clinically compares the tribological behavior and clinical outcome of vitamin E blended HXLPE with ultra-high molecular weight polyethylene (UHMWPE) in an isoelastic monoblock cup for uncemented total hip arthroplasty.

**Patients and methods** — In this randomized controlled trial (RCT), 199 patients were included: 102 patients received the vitamin E blended HXLPE cup, 97 patients the UHMWPE cup. Clinical and radiographic parameters were obtained preoperatively, directly postoperative and at 3, 12, and 24 months. Wear rates were compared using the mean linear femoral head penetration (FHP) rate.

**Results** — 188 patients (94%) completed the 2-year follow-up. Mean patient satisfaction was higher in the vitamin E blended HXLPE group (8.9 [1]) than in in the control group (8.5 [2], p = 0.03). The Harris Hip Score (HHS) was higher in the vitamin E blended HXLPE group (95 [8]) than in the control group (92 [11], p = 0.3). The FHP rate was lower in the vitamin E blended HXLPE group: 0.046 mm/year compared with 0.056 mm/year in the control group (p = 0.05). No adverse reactions associated with the clinical application of vitamin E blended HXLPE were observed during follow-up, with an excellent 2-year survival to revision rate of 98% for both cups.

**Interpretation** — This study shows the superior performance of the HXLPE blended with vitamin E acetabular cup with lower linear femoral head penetration rates and better clinical results compared with the UHMWPE acetabular cup after 2 years.

Periprosthetic osteolysis and aseptic loosening, secondary to wear of the conventional UHMWPE in the acetabular cup, may limit the survivorship of total hip arthroplasty (Willert et al. 1990, Sochart 1999, Harris 2001). Highly cross-linked polyethylene (HXLPE) was introduced in the late 1990s as a response to the high wear rates of UHMWPE causing aseptic loosening and subsequent revision surgery (Kurtz et al. 2011).

The use of HXLPE in THA has been shown to reduce wear significantly (Muratoglu et al. 2001b, Martell et al. 2003b, Bragdon et al. 2005, Snijders et al. 2020). However, cross-linking of polyethylene through the use of ionizing radiation forms free radicals, which leads to a decrease of long-term oxidative stability, causing embrittlement of the HXLPE (Sutula et al. 1995, Collier et al. 1996). Adding an antioxidant, like vitamin E, to the polyethylene might diminish this process (Burton and Ingold 1981, Oral et al. 2004). Several studies have shown good in-vitro and -vivo results of the vitamin E stabilized HXLPE in terms of wear rates and mechanical properties (Halma et al. 2015, Nebergall et al. 2017, Scemama et al. 2017, Zijlstra et al. 2017, Lambert et al. 2019). Besides, vitamin E blended HXLPE cups fit larger femoral head sizes due to a thinner liner, which could reduce the risk of dislocation (Zijlstra et al. 2017). However, no results of a large randomized controlled trial (RCT) with vitamin E blended HXLPE cups are available yet. This RCT compares wear rates between the uncemented vitamin E blended HXLPE acetabular cup and the UHMWPE acetabular cup after 2 years of follow-up. The primary outcome is the linear femoral head penetration (FHP) rate as a measure of wear. Secondary outcomes are the effect of increased head size, clinical performance, and complication rate between the 2 cups.  

## Patients and methods

### Study design

This single-blinded RCT was carried out at the Diakonessenhuis Hospital, a medium-size general hospital in Utrecht/Zeist, the Netherlands. Between 2011 and 2014, 199 patients were included, and randomly allocated in 2 groups. Allocation of patients was done using an internet randomization system (ALEA, FormsVision, Abcoude, The Netherlands).

After randomization, 102 patients received an uncemented vitamin E blended HXLPE cup (RM uncemented monoblock pressfit Vitamys cup, Mathys Ltd, Bettlach, Switzerland) and 97 received a conventional UHMWPE cup (RM uncemented monoblock pressfit, Mathys Ltd, Bettlach, Switzerland).

Baseline characteristics and indications for THA are given in Table 1. All patients scheduled for primary THA aged between 20 and 85 years with primary osteoarthritis, osteoarthritis due to hip dysplasia, rheumatoid arthritis, avascular necrosis of the femoral head, or trauma, and willing to participate, were eligible. Patients with an ASA score ≥ 3 were excluded. After enrollment, baseline characteristics, Harris Hip Score (HHS), numeric rating scale (NRS) score for rest and load pain, and patient satisfaction were documented.

**Table 1.** Demographics. Values are n (%) unless otherwise specified

		Vitamin E	
	Total	HXLPE **^a^**	UHMWPE **^b^**
Factor	n = 199	n = 102	n = 97
Age, mean (SD)	65 (5)	66 (5)	65 (5)
Weight, mean (SD)	79 (13)	78 (13)	79 (14)
Female sex	141 (71)	77 (75)	64 (66)
Diagnosis			
Primary osteoarthritis	191 (96)	98 (96)	93 (96)
Secondary osteoarthritis	2 (1)	1 (1)	1 (1)
Inflammatory arthritis	1 (1)		1 (1)
Femoral head avascular necrosis	1 (1)		1 (1)
Hip dysplasia	4 (2)	3 (3)	1 (1)

**^a^**Vitamin E diffused highly cross-linked polyethylene.

**^b^**UHMWPE: ultra-high molecular weight polyethylene.

Patients were scheduled for clinical and radiological follow-up on the first day postoperatively, and at 3, 12, and 24 months. At each follow-up HHS and NRS score, as well as complications or adverse reactions, were documented and anteroposterior radiographs (AP) of the pelvis in supine position were taken. Follow-ups were performed by 2 independent investigators who were blinded for the intervention (authors JvE and TS). In order to reduce bias, the patient was blinded and the surgeon did not perform the follow-up measurements. The endpoint of this study was set at 6 years’ follow-up or any case of revision or death.

### Sample size calculation

An RCT by Dorr et al. (2005) found a lower mean wear rate at 2 years in the group treated with the HXLPE cup (0.150 mm [SD 0.09]) than in the group with an UHMWPE cup (0.191 mm [SD 0.09]). Sample size calculation was based on a mean difference of wear rate of 0.040, with a standard deviation of 0.09 mm. The sample size was determined on the basis of an overall α-error of 0.05 and a statistical power of 80% (ß = 0.20). This resulted in a sample size of 80 patients per group, which was increased to 100 patients per group to account for possible withdrawal and loss-to-follow-up.

Subjects were able to leave the study at any time or reason, without any consequences. The investigators were able to withdraw a subject from the study for urgent medical reasons. There was no replacement of subjects after withdrawal.

### Procedure

Critical aspects of the surgical procedure were standardized for both groups. Surgeons required knowledge of the allocated treatment, and therefore were not blinded for the intervention. They were informed about the cup type during surgery. The procedures were performed by 7 orthopedic surgeons, each with vast experience in uncemented THA. Alumina ceramic femoral prosthetic heads (BIONIT2, Mathys Ltd, Bettlach, Switzerland) of 28, 32, or 36 mm were used. An uncemented hydroxyapatite coated stem (Twinsys, Mathys Ltd, Bettlach, Switzerland) was implanted in all cases.

Key aspects of pre- and postoperative care were protocolled in order to ensure similar perioperative regimens. All patients received cefazolin prophylaxis during the 24 hours perioperatively and thromboprophylaxis with low molecular weight heparin for 6 weeks postoperative. Both groups followed the same rehabilitation regimen, starting on the first day after surgery.

### Radiological assessment

Radiological assessment was performed by 1 of the authors (JM) according to a standardized form. The inclination of the acetabular component was measured. If present, then the radiolucency and osteolysis around the cup and stem and the affected zone, as described by DeLee and Charnley (1976), was scored. The Brooker classification was used for grading heterotopic ossifications (Brooker et al. 1973).

The FHP measurements were performed on a PACS workstation with a high-resolution monitor using View Pro-X software version 4.0.8.9 (Rogan-Delft, Veenendaal, The Netherlands). All measurements were performed using a computer-assisted method for measuring the FHP of all-polyethylene cups (modified dual-circle technique) previously described by Geerdink et al. (2008). AP pelvic radiographs were calibrated using the size of the prosthetic femoral head as reference. The FHP rate in mm/year was calculated by subtracting the distance measured at 2 years’ follow-up from the distance measured at 1 year and subsequently divided by a correction for the follow-up interval in days. Furthermore, the total FHP was measured by subtracting the distance measured at 24 months from the distance measured on the first day postoperatively, at 3 months, or at 12 months and subsequently divided by the correction for the follow-up interval in days.

### Statistics

Statistical analysis was performed using SPSS Statistics, version 23.0 (IBM Corp., Armonk, NY, USA). The distribution of the data was checked using the Kolmogorov–Smirnov test. The Pearson chi-square test was used to test for significant differences between groups in head size, surgical approach, and radiographic specifics (Brooker classification, radiographic lucency around the cup and stem, and osteolysis around the stem). Furthermore, multivariable analyses were used to test for significant relationships of these variables with the FHP rate within 1 group. A paired t-test was used to test for significant differences in both groups between the FHP rate in the first year and in the second year. An independent t-test was used to test for significant differences in FHP rate between the two groups.

### Ethics, registration, data sharing, funding, and potential conflict of interests

The procedures performed in this study, involving human participants, were in accordance with the ethical standards of the institutional and/or national research committee, with the 1964 Declaration of Helsinki and its later amendments or comparable ethical standard and the CONSORT statement. All patients gave informed written consent. The protocol was approved by the local institutional review board and registered at Central Commission Human-Related research (CCMO) Registry as HipVit trial (NL 32832.100.10, R-10.17D/HIPVIT 1). Data are available from the corresponding author on reasonable request. This study was funded by the Clinical Orthopedic Research Foundation, Diakonessenhuis Zeist. The authors declare no relevant conflict of interest.

## Results

**Figure 1.**
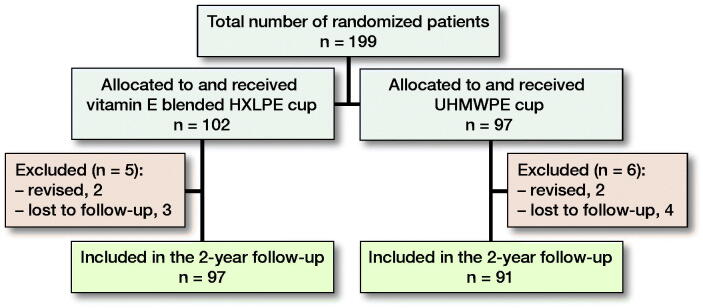
Summary of follow-up. 1 patient had died due to unrelated diseases, 4 patients were untraceable, and 2 patients left the study because of severe comorbidity. Furthermore, radiographical data were unavailable for 3 patients in the conventional HXLPE group and 5 in the vitamin E blended HXLPE group.

188/199 patients (94%) completed the 2-year follow-up. Patient characteristics and surgery specifics are presented for both groups (Tables 1 and 2). 7 patients (4%) were lost to follow-up (Figure 1). 4 patients (2%) were excluded because they underwent revision surgery. In the vitamin E blended HXLPE group 2 patients underwent revision surgery: 1 for a deep infection and 1 for recurrent instability. In the UHMWPE group, 2 patients underwent revision surgery: 1 for cup malpositioning and 1 for recurrent instability.

**Table 2.** Surgery specifics. Values are n (%)

		Vitamin E	
	Total	HXLPE **^a^**	UHMWPE **^b^**
Factor	n = 199	n = 102	n = 97
Approach			
Direct lateral	98 (49)	45 (44)	53 (55)
Posterolateral	85 (43)	46 (45)	39 (40)
Anterolateral	16 (8)	11 (11)	5 (5)
Head size			
28 mm	22 (11)	2 (2)	20 (21)
32 mm	112 (56)	35 (34)	77 (79)
36 mm	65 (33)	65 (64)	–
Inclination			
< 35°	16 (8)	5 (5)	11 (11)
35–40°	47 (24)	20 (19)	27 (28)
41–45°	54 (27)	26 (26)	28 (29)
46–50°	51 (26)	31 (30)	20 (21)
> 50°	31 (16)	20 (20)	11 (11)

**^a, b^**See Table 1.

The head sizes used in the vitamin E blended HXLPE group were larger than the head sizes in the control group (p < 0.001). Bivariate analysis showed no statistically significant differences in heterotopic ossifications (p = 0.4), radiographic lucency (p = 0.3), or osteolysis (p = 0.8) around the stem. The vitamin E blended HXLPE cups showed less radiographic lucency around the cup (p = 0.04). The mean time to the 2-year follow-up visit was 27 (SD 5.4) months.

### Femoral head penetration

**Figure 2.**
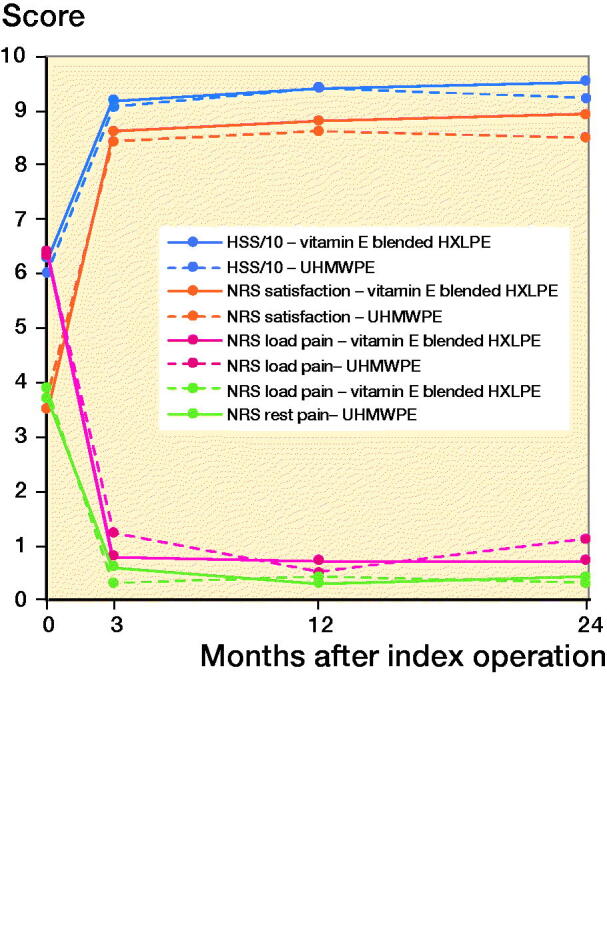
Total femoral head penetration (FHP) in mm and mean FHP rate in mm/year.

The FHP rates were normally distributed. The total FHP after 2 years was 0.27 mm in the vitamin E blended HXLPE cup and 0.28 mm in the UHMWPE cup (Table 3). The FHP rate from 1 to 2 years, thus excluding the bedding-in time of 1 year, was 0.046 and 0.056 mm/year for the vitamin E blended HXLPE cup and UHMWPE cup respectively (Figure 2).

**Table 3.** Femoral head penetration rate (mm). Values are mean (SD) [95%CI]

Follow-up (months)	Vitamin E		
	HXLPE **^a^**	UHMWPE **^b^**	p-value ^c^
0–3	0.16 (0.1) [0.15–0.19]	0.16 (0.1) [0.13–0.18]	0.5
0–12	0.24 (0.1) [0.21–0.27]	0.25 (0.1) [0.22–0.27]	0.9
12–24	0.046 (0.03) [0.040–0.052]	0.056 (0.04) [0.048–0.065]	0.05
0–24	0.27 (0.1) [0.25–0.30]	0.28 (0.1) [0.26–0.31]	0.8

**^a, b^**See Table 1.

**^c^** p < 0.05 = statistically significant.

An independent t-test showed a significant lower FHP rate of the vitamin E blended HXLPE cup after 2 years (p = 0.05) (Table 3). FHP rates for each head size in both groups are given in Table 4, see Supplementary data). There was a statistically significant difference between the FHP rate in the first and second year in both groups (vitamin E blended HXLPE cup: p < 0.001, UHMWPE cup: p < 0.001). There were no statistically significant differences in wear rates between head sizes of same cup type (Table 4, see Supplementary data).

### Clinical outcomes

**Figure 3.**
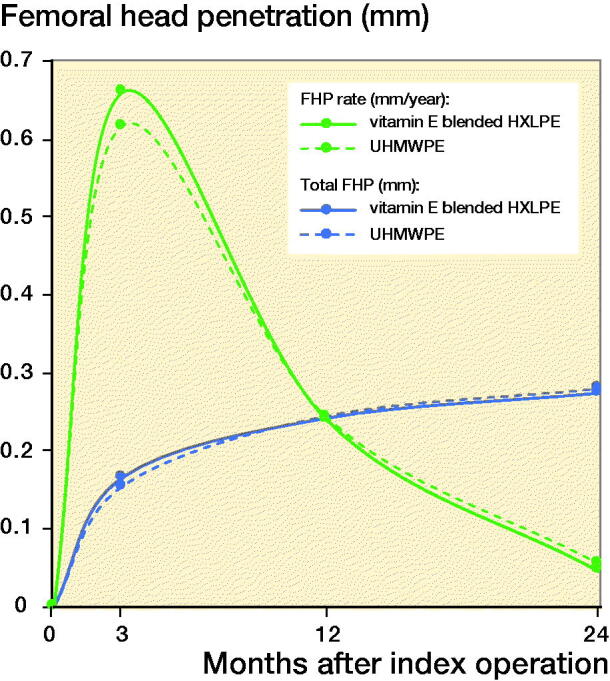
Harris Hip Scores (HHS) and Numeric Rating Scale (NRS) scores.

NRS and Harris Hip scores at baseline and at each follow-up are presented in Figure 3. The scores for both groups at 2-year follow-up are specified in Table 5. The HHS was higher in the vitamin E blended HXLPE group (p = 0.03), as was the NRS score for patient satisfaction (p = 0.03). Specified results for both groups at baseline, 3-, and 12-months’ follow-up can be found in Tables 6, 7, and 8, see Supplementary data. The NRS scores for rest and load pain were not significantly different between groups (p = 0.6, p = 0.06).

**Table 5.** Numeric Rating Scale (NRS) and Harris Hip Scores (HHS) at the 2-year follow-up. Values are mean (SD)

		Vitamin E		
	Total	HXLPE **^a^**	UHMWPE **^b^**	
Factor	n = 180	n = 94	n = 87	p-value ^c^
NRS rest pain	0.4 (1)	0.3 (1)	0.4 (1)	0.6
NRS load pain	0.9 (2)	0.7 (2)	1.1 (2)	0.06
NRS satisfaction	8.7 (1)	8.9 (1)	8.5 (2)	0.03
HHS	94 (10)	95 (8)	92 (11)	0.03

**^a, b^**See Table 1.

**^c^** p < 0.05 = statistically significant.

### Complications

No adverse reactions associated with the clinical application of vitamin E-blended HXLPE were observed. In 2 patients with a vitamin E blended HXLPE cup, a perioperative fracture of the femur occurred. In one patient with a UHMWPE cup, a postoperative fracture of the acetabulum occurred. All patients were treated nonoperatively and recovered completely without further complications.

Within the 2 years’ follow-up 13 complications occurred (Table 9, see Supplementary data). All patients recovered completely, except for 2 patients who continued to have mild paresthesia, caused by neuropraxia of the sciatic nerve.

The 2-year survival to revision rate is 98% for both groups. The 2-year survival rate for aseptic loosening is 100% in both groups.

## Discussion

This RCT is the first to compare 2 similar uncemented acetabular monoblock cups in terms of wear rates; one made of vitamin E blended HXLPE and one of conventional UHMWPE. The vitamin E blended HXLPE showed a lower (p = 0.05) FHP rate of 0.046 mm/year compared with the conventional HXLPE cup (0.056 mm/year). No adverse reactions or abnormal mechanical behavior, concerning the clinical application of vitamin E, were observed after 2 years postoperatively.

Previous in-vitro and -vivo studies have shown that vitamin E blended HXLPE has improved wear rates and protects against oxidation and material embrittlement (Halma et al. 2015, Nebergall et al. 2017, Scemama et al. 2017, Zijlstra et al. 2017, Lambert et al. 2019). The vitamin E blended HXLPE showed a statistically significant superior mean linear FHP rate of 0.046 mm/year compared with the conventional HXLPE cup (0.056 mm/year). Many studies show that a substantial amount of the FHP occurs within the first year of a component being in vivo, due to bedding-in of the liner or creep of the polyethylene (Sychterz et al. 1999, McCalden et al. 2005). Therefore, we attributed the first year of linear FHP to the creep of the vitamin E blended HXLPE liner (Rochcongar et al. 2018, Snijders et al. 2020). The total FHP in our study was 0.27 mm in the vitamin E blended HXLPE cup and 0.28 mm in the UHMWPE cup. This is within the range of results reported in current literature (Salemyr et al. 2015, Shareghi et al. 2015). After the bedding-in time, a steady-state penetration rate is reached. Our study showed a statistically significant difference between the FHP rate in the first and second year in both groups, suggesting that steady-state penetration has not been reached yet. Therefore, at this point, it is impossible to differentiate between bedding-in and actual wear.

Several studies have already shown superior performance of vitamin E addition to HXLPE for protection against oxidation, preserving mechanical properties, with reduced wear debris and bacterial adhesion compared with the conventional UHMWPE cups, suggesting that the addition of vitamin E may prevent osteolysis, implant loosening, and eventually revision surgery (Scemama et al. 2017, Yamamoto et al. 2017, Rochcongar et al. 2018, Lambert et al. 2019). A cohort study by Halma et al. (2015) measured a similar mean FHP rate of a vitamin E blended HXLPE cup of 0.055 mm/year in 107 patients after 2 years. A small RCT by Rochcongar et al. (2018), comparing the same cups in 62 patients, found a statistically significant difference (p < 0.001) between the steady-state FHP rate in a vitamin E blended HXLPE cup (0.020 mm/year) and a UHMWPE cup (0.058 mm/year) after 3 years of follow-up, using radiostereometric analysis (RSA). The slightly lower wear rates for the vitamin E blended HXLPE cup in the study by Rochcongar et al. could be explained by the extended period of 3 years’ follow-up. Due to the extended follow-up, the influence of creep on the wear will reduce and this results in lower measured wear rates. Considering that our short-term study has a mean follow-up time of 2 years and that steady state might not have been reached yet, the wear rate is expected to decrease further in the long term.

We observed no adverse reactions or abnormal mechanical behavior, concerning the clinical application of vitamin E blended HXLPE in an isoelastic monoblock cup for uncemented THA, after 2 years postoperatively. This is consistent with 2 clinical studies, which show no adverse reactions at intermediate follow-up (Halma et al. 2015, Snijders et al. 2020). Therefore, clinical application of vitamin E blended HXLPE could be considered safe. Yet, uncertainties remain about the long-term application of vitamin E.

This study has several limitations. First, only a linear, 2-dimensional, penetration measurement was performed, using the View Pro-X software instead of the 3D RSA measuring method (Callary et al. 2015). Nevertheless, this software has been found to be reliable in clinical practice for the assessment of linear FHP to determine the polyethylene wear, with good inter- and intra-class reliability (Martell et al. 2003a, Geerdink et al. 2008). Second, patients in the vitamin E blended HXLPE group received larger head sizes, but, similar to other studies, multivariate analysis showed no statistically significant different effect of head size on the wear in both cups (Lachiewicz et al. 2009, 2016) (Table 4, see supplementary data). Some studies found larger head sizes to cause more wear (Jasty et al. 1997, Cooper and Della Valle 2014). Therefore, the larger head sizes in the vitamin E group can only cause an underestimation of the effect. However, the marked reduction in wear with the introduction of HXLPE has diminished this concern (Muratoglu et al. 2001a, Estok et al. 2007, Cooper and Della Valle 2014).

Furthermore, our study shows statistically significant superior clinical results for the vitamin E blended HXLPE cup compared with the UHMWPE cup. However, both cups show excellent outcomes in terms of NRS and HHS score. Therefore, these results are not considered to be clinically significant and could be influenced by the greater head size of the vitamin E blended HXLPE cup. Due to the availability of the vitamin E blended HXLPE cup on the Dutch market, surgeons only have a choice of larger head sizes in most similar cup sizes. In its manufacturing process thinner polyethylene liners are applied because less wear is expected. This brings the advantage of the potential lower risk of dislocation with the use of larger heads in the vitamin E blended HXLPE cups compared with the UHMWPE cups (Cooper and Della Valle 2014).

In conclusion, this RCT shows excellent and superior short-term results of the vitamin E blended HXLPE compared with a UHMWPE control group in terms of wear rate. Further follow-up is needed to assess whether the protection against oxidation due to the vitamin E results in lower wear rates and subsequently less osteolysis and aseptic loosening, compared with conventional UHMWPE in the long term.

## Supplementary Material

Supplemental Material

## Supplementary data

Tables 4 and 6–9 are available as supplementary data in the online version of this article, http://dx.doi.org/10.1080/ 17453674.2020.1730073
