# Supplementary material for: 2-year results of an RCT of 2 uncemented isoelastic monoblock acetabular components: lower wear rate with vitamin E blended highly cross-linked polyethylene compared to ultra-high molecular weight polyethylene
Source: Acta Orthop. 2020 Feb 26;91(3):254–9. doi: 10.1080/17453674.2020.1730073 (PMC8023900; doi:10.1080/17453674.2020.1730073)
Supplement: Supplemental Material [file IORT_A_1730073_SM9223.pdf]

Supplementary data

Table 4. Femoral head penetration rate (SD) (mm/year) per head size during the 1–2-year follow-up

Head size	Vitamin E HXLPE [a]	UHMWPE [b]
28 mm	0.033 (0.02)	0.067 (0.05)
32 mm	0.053 (0.03)	0.054 (0.04)
36 mm	0.043 (0.03)	

[a] Vitamin E diffused highly cross-linked polyethylene.
[b] UHMWPE: ultra-high molecular weight polyethylene.
No significant differences in wear between head sizes of same cup type.

Table 9. Postoperative complications during the 2-year follow-up

Complication	Total n = 199	Vitamin E HXLPE [a] n = 102	UHMWPE [b] n = 97
Cup malpositioning	1	1	
Pulmonary embolism	1	1	
Deep venous thrombosis	1	1	
Neurological damage	2		2
Superficial wound infection	2	1	1
Deep infection	1	1	
Leg length discrepancy	1	1	
Dislocation	4	1	3
Total	13 (7%)	7 (7%)	6 (6%)

[a, b] See Table 4.

Table 6. Numeric Rating Scale (NRS) and Harris Hip Score (HHS) preoperatively. Values are mean (SD)

Score	Total	Vitamin E HXLPE [a]	UHMWPE [b]
NRS rest pain	3.8 (2)	3.9 (3)	3.7 (3)
NRS load pain	6.4 (2)	6.3 (2)	6.4 (2)
NRS satisfaction	3.6 (2)	3.5 (2)	3.7 (1)
HHS	62 (17)	63 (19)	60 (15)

[a, b] See Table 4.

Table 7. Numeric Rating Scale (NRS) and Harris Hip Score (HHS) at 3 months' follow-up. Values are mean (SD)

Score	Total	Vitamin E HXLPE [a]	UHMWPE [b]
NRS rest pain	0.4 (1)	0.3 (1)	0.6 (1)
NRS load pain	1.0 (2)	0.8 (2)	1.2 (2)
NRS satisfaction	8.5 (2)	8.6 (2)	8.4 (1)
HHS	91 (10)	92 (9)	91 (11)

[a, b] See Table 4.

Table 8. Numeric Rating Scale (NRS) and Harris Hip Score (HHS) at the 1-year follow-up. Values are mean (SD)

Score	Total	Vitamin E HXLPE [a]	UHMWPE [b]
NRS rest pain	0.4 (1)	0.4 (1)	0.3 (1)
NRS load pain	0.6 (2)	0.7 (2)	0.5 (2)
NRS satisfaction	8.7 (1)	8.8 (1)	8.6 (1)
HHS	94 (9)	94 (9)	94 (10)

[a, b] See Table 4.
